# Flexibler Entscheidungsalgorithmus zum innerklinischen stationären Patient*innen-Management unter wechselnden Anforderungen der SARS-CoV-2-Pandemie

**DOI:** 10.1007/s10049-020-00748-x

**Published:** 2020-07-07

**Authors:** C. Waydhas, I. Hosbach, S. Bockelmann-Jung, U. Hamsen, P. Kaufmann, V. Knop-Hammad, R. Köditz, J. Kronsbein, P. Zahn, T. Auhuber, Pandemiegruppe des BG-Universitätsklinikums Bergmannsheil Bochum, Thomas Auhuber, Sarah Bockelmann-Jung, Dirk Buchwald, Carsten Eichhorn, Peter Godau, Michael Gottschlich, Uwe Hamsen, Martin Heyer, Ingolf Hosbach, Peter Kaufmann, Veronika Knop-Hammad, Roland Köditz, Juliane Kronsbein, Mareike Lobreier, Alina Renelt, Michael Sichelschmidt, Anke Vonderstein, Christian Waydhas, Jens-Oliver Wengeler, Peter Zahn

**Affiliations:** BG-Universitätsklinikum Bergmannsheil Bochum, Bürkle-de-la-Camp-Platz 1, 44789 Bochum, Deutschland

**Keywords:** Patientenmanagement, Notaufnahme, COVID-19, Algorithmus, Entscheidungsfindung, Patient management, Hospital admission, COVID-19, Algorithm, Decision making

## Abstract

Die nunmehr zunehmend verhalten verlaufende SARS-CoV-2-Pandemie stellt hohe Anforderungen an alle involvierten Kliniken hinsichtlich der Rückkehr zur Elektivversorgung wie auch in Hinsicht auf die Planungen für ein Worst-case-Szenario einer zweiten Welle, z. B. nach wieder vermehrter Reisetätigkeit im Sommer oder im Rahmen wieder zunehmender Erkältungskrankheiten im Herbst. Sowohl für die Erstsichtung und die Notaufnahme als auch innerklinisch ist nach Aufnahme des Patienten flexibel zu reagieren. Deshalb war es unser Ziel, eine Handlungsanweisung in Form eines Algorithmus zu entwickeln und zu implementieren, die den ärztlichen und Pflegemitarbeitern einen verlässlichen Entscheidungsrahmen an die Hand gibt und gleichzeitig Ressourcen schont, Patientenfehlallokationen minimiert und Patient*innen und Personal gleichermaßen Sicherheit und Rückhalt gibt. In dem Algorithmus sind wichtige Entscheidungsknoten wie klinische Symptomatik, Erstkontakt, Übergang in die stationäre Behandlung, Vorgehen nach Abstrich- und Computertomographie (CT)-Ergebnis sowie Allokations- und Isolationsmaßnahmen zusammengefasst. Der Algorithmus soll nicht das initiale rein medizinische Management in der Notaufnahme abbilden, sondern entspricht einem allgemeinen, organisatorischen Prozedere für die stationäre Aufnahme und den Gesamtklinikaufenthalt.

## Abstract (en)

After the initial fulminant outbreak, the SARS-CoV‑2 pandemic has now taken a more protracted course which, nevertheless, challenges hospitals in returning to a “normal” mode and in preparing for a worst-case scenario of a second wave. Not only the organization of the first contact with the patient and the admission in the emergency department but also the admission as an in-patient and the subsequent management requires both flexibility and clear directions of action for the medical personnel involved. The aim of the algorithm was to develop a structured, easy to implement and easy to follow guideline while simultaneously preserving resources. The algorithm covers some key points of decision making such as clinical signs, first contact, admission for in-patient treatment, consequences of swab and computed tomography (CT) results, and allocation and isolation measures within the hospital. The algorithm is not intended to guide diagnostics, decisions and treatment in the narrower medical sense but to provide more general instructions for the management of in-patients considering specific aspects of SARS-CoV‑2.

## Einleitung

Die initial lokal z. T. rasant und nunmehr verbreitet verhalten verlaufende SARS-CoV-2-Pandemie stellt hohe Anforderungen an alle involvierten Kliniken hinsichtlich der Rückkehr zur Elektivversorgung wie auch in Hinsicht auf die Planungen für ein Worst-case-Szenario einer zweiten Welle, z. B. nach wieder vermehrter Reisetätigkeit im Sommer oder im Rahmen wieder zunehmender Erkältungskrankheiten im Herbst. Neben der Vorhalte von Verbrauchsmaterialien und Medikamenten stoßen wünschenswerte Planungswege für die meisten Kliniken auf begrenzte Ressourcen. Dazu zählen v. a. bauliche und personelle Aspekte: Das langfristige Vorhalten von Räumen und Personal mit möglichst strikter Trennung von infektiologischer und nichtinfektiologischer Patientenbetreuung stößt häufig auf eine weniger ideale Realität. Umso größer ist die Bedeutung eines schlanken und flexibel reagierenden Patientenmanagements unter Nutzung der vorhandenen Ressourcen vom Moment des ersten Patientenkontakts bis hin zur Entlassung bzw. Verlegung. Im Zuge eines sich normalisierenden Elektivprogrammes und je nach den Lockerungsmaßnahmen der jeweiligen Landesregierungen wird die Erstsichtung ambulanter wie auch stationär aufzunehmender Patient*innen wieder zunehmend dezentraler. Umso wichtiger ist die einheitliche Steuerung der innerklinischen Patientenströme trotz Dezentralisierung der aufnehmenden Einheiten (Notfallaufnahme, Ambulanzen, Sprechstunden, Aufnahme-Stationen etc.). Auch innerklinisch ist nach Aufnahme u. U. flexibel zu reagieren, da jede Klinik im Laufe der Zeit auch auf SARS-CoV-2-positiv getestete Beschäftigte wie auch Besucher*innen und entsprechende Kontaktpersonen wird reagieren müssen.

Deshalb war es unser Ziel, eine Handlungsanweisung in Form eines Algorithmus zu entwickeln und zu implementieren, die den ärztlichen und Pflegemitarbeitern einen verlässlichen Entscheidungsrahmen an die Hand gibt und die gleichzeitig Ressourcen schont, Patientenfehlallokationen minimiert und Patient*innen und Personal gleichermaßen Sicherheit und Rückhalt gibt.

## Aufnahme und dynamische Festlegung des innerklinischen Wegs

Das stationäre Aufnahmemanagement unter Pandemiekautelen umfasst sowohl fußläufige Patient*innen als auch solche, die vom Rettungsdienst oder im Rahmen von Zuverlegungen aus anderen Krankenhäusern eingeliefert werden. Dies stellt die aufnehmenden Bereiche, darunter häufig die zentralen Notaufnahmen, vor große Herausforderungen, diejenigen Patient*innen zu identifizieren, die möglicherweise eine symptomlose Besiedelung mit SARS-CoV‑2 aufweisen, möglicherweise bereits an COVID-19 erkrankt sind oder als unkritisch im Sinne einer „Coronaerkrankung“ angesehen werden können. Während die Einschätzung von Patient*innen, bei denen respiratorische Symptome zum Aufsuchen der Notaufnahme führen, vergleichsweise klar vorgegeben ist, stellen Patient*innen, die aufgrund einer Verletzung oder einer anderen Erkrankung zur Aufnahme kommen, eine größere Herausforderung dar, um eine Komorbidität hinsichtlich COVID-19 oder asymptomatischer Besiedelung mit SARS-CoV‑2 auszuschließen. Entsprechend kann das Aufnahmeprozedere in drei Phasen eingeteilt werden:

1. Erstsichtung: Gibt es offenkundige Hinweise für eine COVID-19-Problematik [1]? Diese zunächst grobe Einteilung bedingt den weiteren Erstweg der Patient*innen innerhalb der Aufnahmeeinheit im COVID- oder Nicht-COVID-Bereich. Insbesondere für die Aufnahmen via Notfallaufnahme sind parallel die allgemeinen Kriterien von Behandlungsdringlichkeit und -aufwand bei der Ressourcenplanung zu berücksichtigen, z. B.: Reicht ein Untersuchungs- oder Behandlungsraum oder ist ein Schockraum vonnöten?
2. Je nach Zustand der Patient*innen, den jeweiligen föderalen Vorschriften, lokalen Labor- und Raumkapazitäten und Fortschreiten der technischen Diagnostikmöglichkeiten kann ergänzend zur primären COVID-Entscheidung eine Schnelltestung mit Zwischenquarantänisierung innerhalb der Aufnahmeeinheit treten. Dabei ist kritisch anzumerken, dass je nach weiterem Verlauf der Pandemie aufgrund der Mengenverhältnisse zwischen COVID- und Nicht-COVID-Patient*innen der Zuverlässigkeit von Schnelltests insbesondere hinsichtlich falsch-negativer Befunde eine enorme Bedeutung zukommen wird. Andernfalls steht zu befürchten, dass insbesondere bei ausgelasteten Ressourcen (=Einsatz auch unerfahrener Beschäftigter) sich auf eine trügerische Sicherheit verlassen wird und dem folgenden Punkt weniger Beachtung geschenkt wird.
3. Die erweiterte ärztliche Anamneseerhebung, körperliche Untersuchung [1] und ggf. apparative Diagnostik, z. B. CT Thorax [2, 3], muss dann zu einer bereits verfeinerten Arbeitsdiagnose führen, die den weiteren Weg außerhalb der Aufnahmeeinheit bedingt.

Ab diesem Moment muss eine entscheidende Routine des ständigen Hinterfragens der vorher gestellten COVID-Entscheidung einsetzen, welche erst enden kann, wenn die Patient*innen entlassen werden, da auch nach Ablauf der SARS-CoV-2-Inkubationszeit durch die innerklinischen Kontakte jederzeit eine Änderung eintreten kann. Dabei kann nicht genug betont werden, dass die gestern negativ auf SARS-CoV‑2 getestete Patientin heute positiv und morgen infektiös für andere sein kann. Deswegen ist es kennzeichnend für diese Methodik, dass sie für alle klinischen Bereiche gilt und nicht bestimmten Örtlichkeiten zugeordnet werden kann.

Im Rahmen der gesamten Behandlung ist somit immer wieder eine Reihe von Entscheidungen erforderlich:

- Weisen Patient*innen COVID-19-verdächtige Symptome auf?
- Muss ein Abstrich abgenommen werden?
- Welche Isolations- und Hygienemaßnahmen sind erforderlich?
- Auf welcher Station/in welchem Bereich sollen die Patient*innen stationär weiter behandelt werden?
- Wie lange müssen im weiteren Verlauf ggf. begonnene Isolationsmaßnahmen fortgeführt werden?

## Patienten- und Beschäftigtenschutz

Für Aufnahmeeinheiten jeglicher Art stellt sich immer wieder die Frage nach der einzusetzenden persönlichen Schutzausrüstung (PSA), v. a. beim ersten Kontakt ohne genauere anamnestische Erkenntnisse. Während für COVID-19-Verdachtsfälle die Zusammenstellung vergleichsweise klar geregelt ist [4] und sich nur in Details zwischen Robert-Koch-Institut (RKI) und Fachverbänden unterscheidet (z. B. FFP2- bzw. FFP3-Masken-Einsatz bei potenziell aerosolprovozierenden Tätigkeiten), trifft das für aus anderen Gründen ein Krankenhaus aufsuchende Patient*innen nicht zu. Häufig stellt sich dann fast automatisch ein Dualismus aus „Schutz von begrenzten Vorräten der Schutzmaterialien“ und „lieber auf Nummer sicher gehen“ ein. Der Wunsch nach einem möglichst hohen Sicherheitsniveau lässt jedoch meist mehrere, z. T. pandemietypische Aspekte außer Acht:

1. Das Ansteckungsrisiko bei bzgl. COVID-19 symptomlosen Patient*innen ist während einer Pandemie gleich hoch wie das Ansteckungsrisiko für alle Mitarbeitenden untereinander. Das Risiko wird im Wesentlichen bestimmt durch die Anzahl der Sozialkontakte und liegt bei den meisten Mitarbeitenden durch die beruflich höhere Anzahl an Sozialkontakten ggf. sogar höher. Daraus folgt, dass das Schutzniveau gegenüber symptomlosen Patient*innen dem entsprechen sollte, was als Schutzmaßnahmen zwischen Mitarbeitenden vorgesehen ist. Besteht z. B. eine allgemeine Pflicht zum Tragen von Mund-Nase-Schutz (MNS) in einer Klinik, so ist dies auch für den Kontakt zu symptomlosen Patient*innen sinnvoll. Je nach Bundesland sehen das auch die Coronaschutzverordnungen vor (z. B. CoronaSchVO NRW vom 30.05.2020, § 2 [5]).
2. Das Tragen von FFP2-, N95- bzw. FFP3-Masken ist auf Dauer belastender als das Tragen von MNS. Dies spielt auch jenseits von Atemwiderstand etc. aufgrund in vielen Kliniken fehlender Klimaanlagen bei steigenden Außentemperaturen eine zunehmend limitierende Rolle. Insofern sollte das Tragen dieser Masken engen Kontakten zu COVID-(Verdachts‑)Fällen vorbehalten bleiben.
3. Der ggf. flächendeckende Einsatz von FFP1-, -2- und -3-Masken (Atemschutzmasken = ASM) bedingt die entsprechend ausgeweitete betriebsmedizinische Angebotsvorsorge nach der berufsgenossenschaftlichen Empfehlung G26 [6], was u. U. zur weiteren Verminderung einsetzbaren Personals führen kann.
4. Selbst eine Ausweitung von ASM auf aerosolprovozierende Tätigkeiten bei symptomlosen Patient*innen hat enorme Folgen für die Maskenressourcen: Letztlich ist nicht nur eine Bronchoskopie und Intubation darunter zu fassen, sondern jegliche Mundpflege, logopädische Behandlung, Hirnnerventestung und Geburtsbegleitung. Insbesondere bei diesem Punkt ist auch eine zeitliche Komponente bei der Entscheidung für oder gegen einen solchen Einsatz zu berücksichtigen: Ist die Versorgung mit ASM auch bei gestiegenem Bedarf langfristig und i. R. einer „zweiten Welle“ gesichert?
5. Die aus arbeitsmedizinischer Sicht diskussionswürdige einseitige Betonung der mechanischen Barriere auch gegenüber infektiösen Flüssigkeiten hat zum pandemieunabhängigen Einsatz von flüssigkeitsdichten Schutzkitteln nach EN 14126 bzw. EN ISO 22610 geführt. Auch bei nur teillaminierten Schutzkitteln führt dies bei den zu erwartenden sommerlichen Temperaturen zu teilweise ernsten Gesundheitsstörungen aufgrund der eingeschränkten Transpiration. Durch die bekannten Lieferprobleme dieser teillaminierten Kittel werden vermehrt auch Schutzkittel komplett aus Folie angeboten und auch eingesetzt, die das Problem der dann komplett fehlenden Transpiration potenzieren. Entsprechend sollten ein entsprechendes Getränkeangebot und kurzfristig intermittierende Pausen eingeplant werden. Insofern sollten bei hinsichtlich COVID-19 unverdächtigen Patient*innen keine bzw. möglichst gering belastende Schutzkittel eingesetzt werden und teillaminierte Kittel nur bei engem Kontakt mit patientenassoziierten Flüssigkeiten. Dies sollte jedoch insbesondere bei Triagepersonal nur in Einzelfällen zutreffen.

Der hier vorgeschlagene differenzierte Einsatz von Persönlicher Schutzausrüstung (PSA) hat in unserem Klinikum bei zz. 108 vorrangig unter NFA-, Quarantäne- und Infektionsstationspersonal durchgeführten Antikörpertests (Euroimmun-Test) in keinem Fall eine berufliche Ansteckung bei zz. 234 kumulativ betreuten COVID-19- bzw. COVID-19-Verdachtsfällen ergeben. Der Eintrag von per PCR erkannten SARS-CoV-2-Fällen ist durch Personal bislang dreimal höher als durch Patient*innen. Nur eine wahrscheinliche Ansteckung von Personal an einem nach Aufnahme positiv gewordenen Patienten trat vor Einführung einer allgemeinen Pflicht zum Tragen von MNS auf, seither sind keine internen Infektionsketten mehr aufgetreten.

Noch vor den Überlegungen zum Einsatz von PSA sollten technische und organisatorische Maßnahmen zur Gefährdungsreduktion stehen. Die betrifft v. a. die Raumkonfiguration und -auswahl:

- COVID-Bereiche sollten möglichst mit Schleusen ausgestattet sein, die groß genug sind, um den ggf. notwendigen materialressourcenschonenden Einsatz beispielsweise von Masken oder/und Mehrwegschutzkitteln zu erlauben.
- Räume der COVID-Versorgung sollten möglichst eng beieinander und ohne Kreuzung der Wege von infektiologischen und sonstigen Patient*innen gewählt werden. Gleiches gilt idealerweise auch für die Wege des eingesetzten Personals in einer Schicht.
- Ideal sind gedoppelte räumliche Einheiten (z. B. jeweils ein Schockraum für COVID-19-Patient*innen und für sonstige Patient*innen). Andernfalls sind umso mehr Ressourcen für eine schnelle und zuverlässige Raumreinigung vorzuhalten.
- Zur weiteren Infektionsgefahrreduzierung sind eine zentrale Überwachung wie auch eine Fernkommunikationseinrichtung mit den Patient*innen von Vorteil.
- Der weitere Transfer von COVID-19-Patient*innen sollte nach den gleichen Maßgaben geplant werden. Insofern macht es ggf. Sinn, zu Aufnahmeeinheiten benachbart liegende Stationen und Aufzüge für die COVID-19-Betreuung zu reservieren.
- Bei jeglicher Raumplanung sollten auch (längere) Verweilzeiten sowohl bei niedrigen wie auch hohen Patientenaufkommen eingeplant werden.
- Aufgrund der Risikoassoziierung mit der Anzahl der Sozialkontakte macht eine Schichtkohortierung des Personals keinen Sinn. Ein „Unter-sich-Bleiben“ führt nur zu trügerischem Sicherheitsgefühl und ist kein Ersatz z. B. für das Tragen eines MNS.

Letztlich zeigt jedoch der idealerweise differenzierte Einsatz von PSA wie auch die Raumzuteilung insbesondere für die weitere Betreuung der Patient*innen auch hier die Bedeutung der innerklinischen Einschätzung hinsichtlich COVID-19.

## Erfassung und Wichtung von Symptomen

Vor allem die Zuordnung der Symptome zu COVID-19 [1] unterliegt einer starken Dynamik sowohl im wissenschaftlichen Erkenntnisprozess als auch im jahreszeitlichen Verlauf hinsichtlich konkurrierender Erkrankungen. So ist beispielsweise der für COVID-19 vergleichsweise untypische Fließschnupfen (<5 %) mit Beginn des Pollenflugs im Frühjahr unabhängig von COVID-19 häufiger geworden und kann allein betrachtet in die Irre führen. Leider geben jedoch Erlasse und Verordnungen das Fragen u. a. nach Schnupfen vor. Auf der anderen Seite wiesen die initialen Daten aus China nicht auf das aktuell wahrscheinlich führende Kardinalsymptom der Anosmie hin, welches wiederum kaum in die amtlichen Erhebungen Eingang gefunden hat. Darüber hinaus ist methodologisch zu beachten, dass im klinischen Alltag v. a. das gefragt und erfasst wird, was bereits vorher als Kardinalsymptom gilt. Andere, möglicherweise wegweisende Symptome können so systematisch untergehen. Dazu könnten gastrointestinale Symptome [7], Hauterscheinungen [8] oder/und Gerinnungsstörungen [9] zählen. Für bestimmte Altersgruppen bestehen Besonderheiten, wie z. B. das Projizieren von Symptomen auf Bauchschmerzen bei jüngeren Kindern wie auch altersspezifische Probleme, wie das „Kawasaki-like syndrome“ [10]. Entsprechend wäre zentral aktualisierte, aber datenschutzkonform dezentral installierte Befragungssoftware mit anonymisierter Auswertefunktion wünschenswert. Das mag zwar epidemiologisch nicht optimal sein, aber ein Bestehen auf zentraler Erfassung von Patientendaten führt in der Praxis aufgrund teilweise berechtigter datenschutzrechtlicher Bedenken zu Verzögerungen und ggf. keinem Einsatz dieser Tools.

In Ermangelung solcher Instrumente wurde insbesondere während der seinerzeit noch laufenden Erkältungs- und Influenzazeit eine ständig aktualisierte Tabelle in den mittlerweile in 45 Versionen fortgeschriebenen Pandemieplan unseres Klinikums aufgenommen, welche nach jeweiligem Kenntnisstand Wichtungen bei Symptomen zwischen grippalen Infekten, Influenza und COVID-19 vornimmt. Dieser dynamische Pandemieplan gilt weiterhin als Dienstanweisung inkl. der Verpflichtung, sich über die Änderungen zur Vorversion zu informieren. Die daraus gewonnenen Erkenntnisse wurden auch in die Fragebögen übernommen, die z. B. ambulante Patient*innen, aber auch Besucher*innen ausfüllen müssen.

Jegliche Erfassung anamnestisch wegweisender Symptome kommt jedoch an ihre Grenzen, wenn bspw. die einzige fremdanamnestische Angabe für eine demenzkranke Bewohnerin aus einem Altersheim ist: „Die spricht nicht mehr wie sonst!“ In diesen Fällen kommt nach der umso wichtigeren körperlichen Untersuchung der weiteren laborchemischen und bildgebenden Diagnostik (insbesondere CT Thorax bei bettlägerigen Patient*innen) eine besondere Rolle zu. Die medienwirksame Messung der Körpertemperatur direkt beim ersten Kontakt führt nach unseren Erfahrungen angesichts des weitverbreiteten Einsatzes von NSAR inkl. Over-the-counter(OTC)-Präparaten häufig in die Irre und kann eher zu einer Vernachlässigung von z. B. Schutzmaßnahmen führen.

## Die Steuerung der Patientenströme mittels Algorithmus

Aber auch jenseits der Frage, welche Symptome COVID-19-typisch sind, gibt es für alle Aspekte Definitionen und Empfehlungen des RKI, von Fachgesellschaften, von Klinikträgern und verschiedenen Abteilungen innerhalb eines Krankenhauses. Sie haben gemeinsam, dass sie sich dynamisch ändern, sich nicht selten zumindest in Details voneinander unterscheiden und häufig weder in Terminologie noch Inhalten und Prozessen aufeinander abgestimmt sind. Bei den handelnden Personen besteht nicht zuletzt deshalb häufig Unsicherheit bezüglich der korrekten Vorgehensweise.

Ein diese Unsicherheiten hinsichtlich der Patientensteuerung möglichst auffangender Algorithmus wurde von den Mitgliedern der Pandemiegruppe unseres Klinikums entwickelt und sollte den o. g. Entscheidungsfragen Rechnung tragen.

**Figure 1:**
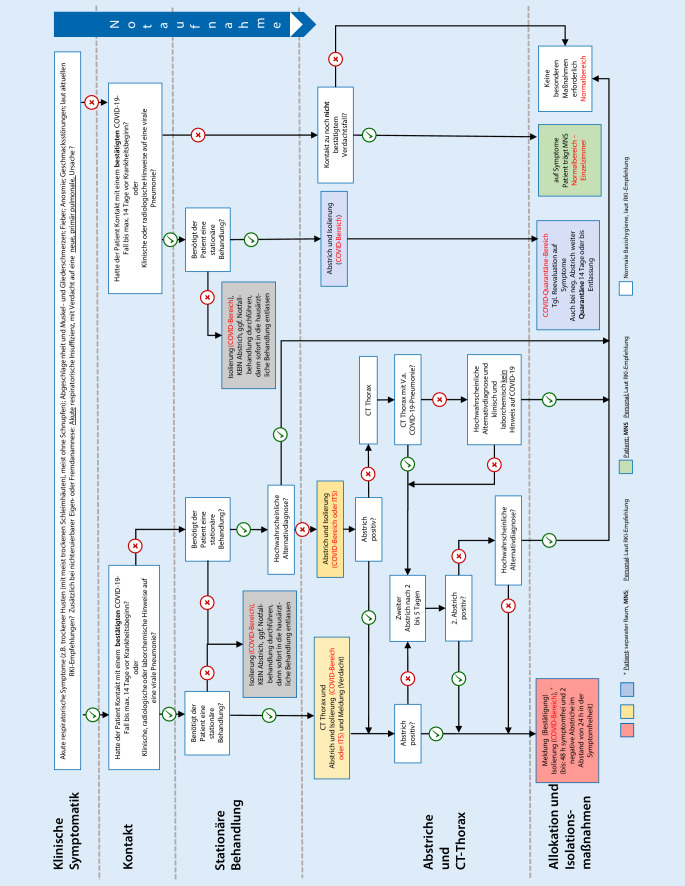

Die erste Version wurde über einen 5‑Tages-Zeitraum an 111 Patient*innen überprüft (Tab. 1). Es zeigte sich, dass der Algorithmus in Bezug auf die Indikation zur Abstrichentnahme nach entsprechender Einweisung unkompliziert umgesetzt und vom Pflege- und ärztlichen Personal in der Notaufnahme und zuarbeitenden Bereichen (Labor, Radiologie etc.) gut akzeptiert wurde. Dies ist der Eindruck, den die Mitglieder der Pandemiegruppe im Rahmen der Begleitung der Prozesse gewonnen haben, ohne dass dies durch eine formale Befragung belegt werden kann. Für die Patientenallokation wurde der Algorithmus (Abb. 1) über mehrere Wochen fortentwickelt und immer wieder hinterfragt. Der Algorithmus wird in Verbindung mit einer standardisierten Anamneseerhebung und Aufnahmetriage (COVID-19-spezifischer Fragenkatalog, ansonsten Manchester Triage Score) angewendet. Beim Initialkontakt zur Erhebung der Anamnese und der Symptome ist sicherer Abstand (z. B. Telefon) zu halten und/oder geeigneter Eigenschutz (mindestens Patienten- plus Beschäftigten-MNS bei fehlenden Erkältungssymptomen bzw. erweiterte PSA bei vorhandenen Erkältungssymptomen) zu beachten.

**Table 1:** 

Initiale Kategorisierung	Patient*innen *n* (%)	1. Abstrich erfolgt (*n*)	1. Abstrich positiv (*n*)
ROT	2 (1,8)	2	2
GELB	6 (5,4)	6	1
BLAU	11 (9,9)	10	2
WEISS	92 (82,9)	3	0
Gesamt	111 (100)	21	5

Vom ersten Kontakt über jegliche neue Veränderung im Ablauf eines Patientenaufenthalts bis zur Entlassung sind mittels des Algorithmus die folgenden Entscheidungsebenen abzuarbeiten und immer wieder zu überprüfen:

1. Entscheidungsebene *klinische Symptomatik* – gibt es klinische Hinweise auf das Vorliegen von COVID-19?
  Die Befragung zu den Symptomen beschränkt sich nicht nur auf die häufigen Symptome, die auf einen viralen oder pulmonalen Infekt hinweisen können (akute respiratorische Symptome, Abgeschlagenheit, Muskel- oder Gliederschmerzen, Fieber), sondern beinhaltet auch COVID-19-typische Beschwerden, wie z. B. eine Anosmie. Schwierig können sich die Anamnese und Symptomerhebung bei bewusstseinsgestörten Patient*innen gestalten, insbesondere wenn auch keine verlässliche Fremdanamnese zu erheben ist. Hier kommt neben der Fremdanamnese und körperlichen Untersuchung der erweiterten laborseitigen und apparativen Diagnostik eine besondere Bedeutung zu. In jedem Fall ist insbesondere am Beginn eines Patientenaufenthalts selten Gewissheit über den COVID-Status von Patient*innen zu erhalten, sondern Wahrscheinlichkeiten prägen den weiteren Weg. Deswegen kommt der kontinuierlichen Reevaluierung eine besondere Bedeutung zu.
2. Entscheidungsebene *Kontakt* – Kontakt mit einem bestätigten COVID-19-Patienten oder klinische oder radiologische Hinweise auf eine virale Pneumonie?
  - Falls diese Frage bejaht wird und die Patient*innen Symptome aufweisen, erfolgen die Isolation der Patient*innen und die Entnahme eines nasalen und pharyngealen Abstrichs. Gleichzeitig wird festgelegt, dass der Patient auf einer Station im „COVID-Bereich“ aufgenommen wird, Intensivstation oder Normalpflegestation je Schweregrad, (Farbcodierung GELB). Weiterhin ist jetzt die Meldung an das Gesundheitsamt (Verdachtsfall) vorzunehmen.
  - Falls diese Frage verneint wird und die Patient*innen weisen Symptome auf, so folgt das gleiche Vorgehen (Farbcodierung GELB), es sei denn, es gibt eine hochwahrscheinliche Alternativdiagnose (z. B. Zuverlegung aus anderem Haus aufgrund eines sekundären ARDS bei einer Sepsis mit bekanntem Fokus). Dann können die Patient*innen „normal“ mit den üblicherweise für die jeweilige Situation erforderlichen Hygienemaßnahmen behandelt werden (Farbcodierung WEISS). Die Entscheidung über das weitere Vorgehen trifft immer die Ärztin/der Arzt vor Ort, gerne auch im Team. Für Zweifels- und Grenzfälle ist es sinnvoll, ein 24/7-Telefon mit pulmonologisch bzw. COVID-19-erfahrenen Kolleg*innen im Hintergrund einzurichten.
  - Falls diese Frage bejaht wird, aber die Patient*innen keine Symptome aufweisen, dann erfolgt die Isolation und die Entnahme von Abstrichen (Farbcodierung BLAU).
  - Falls diese Frage verneint wird und die Patient*innen keine Symptome aufweisen, dann ist zu klären, ob der Patient Kontakt zu einem noch nicht bestätigten Verdachtsfall hatte. In diesem Fall werden keine Abstriche entnommen und die Patient*innen können in Quarantänebereiche verlegt werden (4. Entscheidungsebene, s. unten). Unterschiede gibt es bei den Hygienemaßnahmen. Wenn ein Kontakt mit einem COVID-19-Verdachtsfall bestand, dann erfolgt die Verlegung in ein Einzelzimmer und die Patient*innen tragen einen Mund-Nasen-Schutz (FFP1, MNS; Farbcodierung GRÜN). Bestand kein Kontakt zu einem Verdachtsfall, sind keine besonderen Hygienemaßnahmen in Bezug auf SARS-CoV‑2 erforderlich (Farbcodierung WEISS).
  Die Patient*innen werden im Klinikinformationssystem entsprechend dem Schema markiert. Die Frage nach einem Aufenthalt in einem Risikogebiet spielt dabei aktuell, anders als zu Beginn der Pandemie, kaum eine Rolle mehr.
3. Entscheidungsebene *stationäre Behandlung notwendig?*
  Erfolgte die klinische Überprüfung und Kontaktaufnahme in den (COVID-)Bereichen der Notaufnahme, ist bei stationärer Behandlungsnotwendigkeit der innerklinische Weg zu beschreiben. Bei Patient*innen, die keiner stationären Behandlung bedürfen, erfolgen eine ggf. erforderliche Notfallbehandlung unter Einhaltung der Isolation und persönlichen Schutzmaßnahmen und die Entlassung in die hausärztliche Behandlung, entsprechend den regionalen Anordnungen des Gesundheitsamts. Es werden keine Abstriche entnommen.
4. Entscheidungsebene: *Abstriche und Diagnosesicherung*
  Diese Entscheidungsebene ist nur für Patient*innen mit Symptomen relevant.
  - Im positiven Fall wird die Isolierung fortgeführt, bis die Patient*innen mindestens 48 h symptomfrei sind und 2 negative Abstrichserien (laut den aktuellen Empfehlungen des RKI [11]) im Abstand von mindestens 24 h aufweisen (Farbcodierung ROT) oder die Patient*innen in die ambulante Weiterbehandlung entlassen werden und die weiteren Maßnahmen nach Maßgabe des Gesundheitsamts erfolgen.
  - Bei einem negativen ersten Abstrich trotz klinisch suggestiver Symptome erfolgt ein differenziertes Vorgehen mit den jeweiligen Optionen einer zweiten Abstrichserie und/oder einer Thorax-CT und nach klinischer Beurteilung, um zu prüfen, ob eine hochwahrscheinliche Alternativdiagnose vorliegt. Dies kann dazu führen, dass die Patient*innen in eine andere Kategorie in Bezug auf Isolationsmaßnahmen wechseln und sich die Allokation auf eine andere Station ändert (d. h. von Farbcodierung GELB nach Farbcodierung WEISS).
5. Entscheidungsebene. *Allokation und Dauer der Isolationsmaßnahmen*?
  Am Ende des Algorithmus steht die Empfehlung über die Dauer der Isolationsmaßnahmen und den Bereich innerhalb des Krankenhauses, in dem die Patient*innen während des stationären Aufenthalts behandelt werden. Verdachtsfälle wechseln ggf. in die Kategorie „bestätigte Fälle“ (Farbcodierung ROT).

Kommen im Verlauf der Behandlung neue Informationen hinzu oder treten neue Symptome auf, dann ist der Algorithmus erneut bei der Entscheidungsebene 1 zu beginnen.

Mit dem Algorithmus werden somit die Indikation zur Entnahme von Abstrichen, die Art der einzuleitenden Isolationsmaßnahmen, die Verpflichtung zur Meldung an das Gesundheitsamt, die Festlegung der versorgenden Station (COVID- oder Nicht-COVID-Bereich) und die Dauer der Isolationsmaßnahmen gesteuert. Er folgt den Empfehlungen des Robert Koch-Instituts [1, 11] und muss den jeweiligen Aktualisierungen folgend angepasst werden.

Der Algorithmus weist eine Reihe von Limitationen auf. Er kann und soll nicht dazu dienen, unter Berücksichtigung verschiedener Befundkonstellationen (Labor, Klinik, Bildgebung) die Diagnose zu stellen. Das ist aber auch nicht die Intention des von uns vorgestellten Flussschemas. Er zielt auf das (organisatorische) Management und die Allokation einschließlich der erforderlichen Hygienemaßnahmen ab. Ein diagnostischer Algorithmus ist hier als sinnvolle Ergänzung anzusehen [12, 13]. Wie mit jedem Algorithmus können auch hiermit nicht alle denkbaren Konstellationen eindeutig geregelt werden. So verbleiben Graubereiche in der Wertung von wenig ausgeprägten Symptomen. Auch können Thorax-CT-Zufallsbefunde (z. B. Lungenbefunde bei CT der Schulter oder der Wirbelsäule oder des Abdomens) manchmal schwer eingeordnet werden. Vielmehr wäre dann der Algorithmus beginnend mit der Einstiegsebene nochmals zu durchlaufen. Die Abfrage der Symptome beschränkt sich auf die häufigen und typischen Symptome und Beschwerden. Es gibt jedoch Fälle, bei denen die typischen Symptome (anfangs) fehlen können. Angesichts der großen Zahl unspezifischer Beschwerden ohne COVID-19 und der limitierten Ressourcen erscheint aber eine 100 %ige Sicherheit nicht erreichbar.

Die Stärke des Algorithmus liegt in seiner klaren Strukturierung und darin, dass er die Aspekte Anamnese und Symptome, ein diagnostisches Grundgerüst zum Virusnachweis, angepasste Hygienemaßnahmen, eine Zuordnung zu definierten stationären Bereichen des Krankenhauses (COVID vs. Nicht-COVID) sowie zur Dauer der speziellen Hygienemaßnahmen in einer Synopse berücksichtigt.

Die Ausformulierung von Definitionen, Aufzählungen, Maßnahmen und Erfordernissen im Algorithmus ist nicht als feststehend zu verstehen. Da die medizinischen Erkenntnisse, die Empfehlungen des RKI und auch die gesetzlichen Vorgaben einer ständigen Änderung unterliegen, müssen die Inhalte des Algorithmus daran jeweils angepasst werden.
